# PDGF-B-driven gliomagenesis can occur in the absence of the proteoglycan NG2

**DOI:** 10.1186/1471-2407-10-550

**Published:** 2010-10-12

**Authors:** Marta Terrile, Irene Appolloni, Filippo Calzolari, Roberto Perris, Evelina Tutucci, Paolo Malatesta

**Affiliations:** 1 National Institute for Cancer Research (IST), IRCCS, Largo Rosanna Benzi 10, 16132 Genoa, Italy; 2 Department of Oncology Biology and Genetics (DOBiG), University of Genoa, Largo Rosanna Benzi 10, 16132 Genoa, Italy; 3 COMT - Centre for Molecular and Translational Oncology, University of Parma, Via G.P. Usberti 11/A, Parma 43100, Italy; 4 Laboratory for Stem Cell Research and Cellular Therapy, Division for Experimental Oncology 2, The National Cancer Institute, Aviano, CRO-IRCCS, Via Pedemontana Occidentale 12, Aviano 33081, Italy; 5 Department of Cell Biology, Sciences III, 30 Quai E. Ansermet, 1211 Geneva, 4. Switzerland

## Abstract

**Background:**

In the last years, the transmembrane proteoglycan NG2 has gained interest as a therapeutic target for the treatment of diverse tumor types, including gliomas, because increases of its expression correlate with dismal prognosis. NG2 has been shown to function as a co-receptor for PDGF ligands whose aberrant expression is common in gliomas. We have recently generated a glioma model based on the overexpression of PDGF-B in neural progenitors and here we investigated the possible relevance of NG2 during PDGF-driven gliomagenesis.

**Methods:**

The survival curves of NG2-KO mice overexpressing PDGF-B were compared to controls by using a Log-rank test. The characteristics of tumors induced in NG2-KO were compared to those of tumors induced in wild type mice by immunostaining for different cell lineage markers and by transplantation assays in adult mice.

**Results:**

We showed that the lack of NG2 does not appreciably affect any of the characterized steps of PDGF-driven brain tumorigenesis, such as oligodendrocyte progenitor cells (OPC) induction, the recruitment of bystander OPCs and the progression to full malignancy, which take place as in wild type animals.

**Conclusions:**

Our analysis, using both NG2-KO mice and a miRNA based silencing approach, clearly demonstrates that NG2 is not required for PDGF-B to efficiently induce and maintain gliomas from neural progenitors. On the basis of the data obtained, we therefore suggest that the role of NG2 as a target molecule for glioma treatment should be carefully reconsidered.

## Background

Gliomas are tumors of the central nervous system often characterized by poor survival. Their most aggressive form, glioblastoma, exhibits a dismal prognosis mainly due to its prominent infiltrative ability and to its high radio- and chemo-resistance. Because of this, it is of primary importance to identify molecules that can be used as therapeutic targets. The transmembrane chondroitin sulfate proteoglycan NG2 has been proposed as one of these candidates [1,2].

NG2 is expressed in human gliomas and its expression correlates to their malignancy [3-6] and chemo-resistance [7]. A major role for NG2 in glioma progression could reside in its ability to promote neoangiogenesis [4,8,9] and some reports showed that it could also be involved in other critical processes such as cell migration and cell proliferation [10]. Many of these effects are due to the ability of NG2 to influence extracellular signaling by interacting with integrins [10-12] and growth factor receptors [13-15]. In particular, NG2 was reported to physically interact with PDGF receptor-alpha and its ligand PDGF-A, leading to enhanced PDGF-A signaling activity [13-16]. Importantly, PDGF signaling is commonly altered in human high grade gliomas, which often overexpress PDGF-A or -B ligands or their receptors [17-21]. Furthermore, many studies performed in mouse clearly demonstrate that sustained PDGF-A or -B signaling induces gliomas that closely resemble the human neoplasia [22-30] and are characterized by high NG2 expression levels [24,25,31]. Moreover, we recently demonstrated that PDGF-B overexpression is able to induce NG2 expression in neural precursors [23]. The above data suggest a potential role for the proteoglycan NG2 in gliomagenesis. Notably, even if adult NG2-KO mice do not show any strong phenotype [13,32], it has been reported, though on the basis of a limited sample, that the lack of NG2 could be responsible for a reduction in the incidence of PDGF-B-induced tumors [33] indicating the possibility to disrupt NG2 activity as a way to treat gliomas.

Here, in order to investigate the potential role of this proteoglycan in the formation and progression of PDGF-expressing gliomas, we tested the ability of PDGF-B overexpression to induce brain tumors in NG2-KO mice. The generation of high grade gliomas in this context clearly demonstrated that NG2 is not necessary for the induction and progression of glial tumors, reducing the chances of treating gliomas by inhibiting the function of NG2.

## Methods

### Retroviral vectors

The cDNA of human PDGF-B, derived from the RCAS-pBIG plasmid (kindly provided by Dr. E. Holland, Memorial Sloan-Kettering Cancer Center, New York, USA), was inserted into the SalI site upstream the IRES sequence of the pCEG retroviral backbone (kindly provided by Gordon Fishell, The Skirball Institute of Biomolecular Medicine, New York, USA), generating the PDGF-B/GFP construct. The same PDGF-B cDNA was inserted also into the blunted PmeI/SfiI sites of the pCAG:Ds-Red vector (kindly provided by Dr. M. Goetz, Institute of Stem Cell Research, Germany), upstream the IRES-Ds-Red reporter cassette to form the PDGF-B/Ds Red vector. The engineered miRNA directed against the mRNA of NG2 (the sequence is available on request) was designed using the BLOCK-iT RNAi Designer software of Invitrogen and cloned following the BLOCK-iT™ Pol II miR RNAi Expression Vector kit (Invitrogen) into the gateway cassette (Invitrogen) of the pCDB-GW vector together with the EmGFP cDNA. As negative control we used the miRneg oligo supplied in the kit and we cloned it in the pCDB-GW plasmid. The pCDB-GW vector was derived by the pCEG vector after the deletion of the GFP cDNA and the insertion of the gateway cassette in the SalI site.

Replication-defective retroviral supernatants were prepared by transiently transfecting plasmids into Phoenix packaging cells as described in [34].

### Cell cultures

Primary cultures of embryonic neural precursors were obtained from embryonic day 14 (E14) mouse embryos. Dorsal and ventral regions were separated and kept in HBSS buffer (Invitrogen, Carlsbad, CA) added with 1 mM HEPES (Invitrogen). Forebrain explants were then incubated in 1 ml of trypsin/EDTA (Invitrogen) at 37°C for 15 minutes and then mechanically dissociated with a glass pipette in DMEM medium added with 10% foetal calf serum (Invitrogen). Cells were then plated at a density of 3.5 × 10^5 ^cells/cm^2 ^onto poly-D-lysine (Sigma, St. Louis, MO) coated coverslips. Retroviral supernatants were added immediately after plating. Cells were cultured in a chemically defined medium (SATO medium [35]) and fixed in 4% paraformaldehyde after 6 days.

Tumor cell cultures were established as described in [25]. Briefly, tumor masses were dissected under fluorescence microscope by taking advantage of the expression of GFP and trypsinized for 20 minutes. After trypsin neutralization with serum-containing DMEM medium, cells were resuspended in DMEM-F12 added with B27 supplement, human bFGF (20 ng/ml, Invitrogen) and EGF (20 ng/ml, Invitrogen) and plated on Matrigel-coated flasks (BD Biosciences, San Jose, CA). For the miR-NG2 experiments, tumor cell cultures derived from PDGFB/DsRed induced gliomas were transduced by adding high titre retroviral supernatants one day after cell splitting. Before in vivo injections the percentage of GFP-positive cells was determined counting the cells using a burker chamber under a fluorescent microscope.

### Quantification of cell foci density

For foci formation, cells were allowed to overgrow on 6-well plates and then the average number of foci in 4 mm^2 ^area was determined for two wild type and two NG2-KO glioma cultures. For each well, three random areas were analyzed and the count was repeated for three days to monitor foci formation during time. The experiment was then repeated twice.

### Quantification of infiltrating cells

For infiltrating cell quantification at least two NG2-KO and three wild type gliomas were analyzed. Tumor cells migrated in the healthy brain tissue were identified in brain sections by means of their immunopositivity to GFP. To determine the number of infiltrated cells, brain regions of 0.1 mm^2 ^near the tumor masses were analyzed. The number of infiltrating cells per mm^2 ^was then obtained for each section and used to calculate the average number of infiltrating cells for the two tumor groups (wild type and NG2-KO).

### In vivo injections

Mice were handled in agreement with Italian guidelines and regulations about animal use for scientific purposes (D.lvo 27/01/1992, n. 116). All procedures were approved by the Ethical Committee for Animal Experimentation of the National Institute of Cancer Research and by the Italian Ministry of Health. All experiments were performed on C57BL/6 mice and C57BL/6 NG2 null mice (kindly provided by Dr. William B. Stallcup) which in the text are referred to as wild type and NG2-KO mice, respectively.

*In utero *intraventricular injections were performed on anesthetized pregnant dams at the fourteenth day of gestation. Following laparotomy, uterine horns were exposed and embryos were injected within the telencephalic ventricles with approximately 2 μl of retroviral suspension containing 1% polybrene (Sigma) to facilitate cell infection. After birth, injected animals were daily monitored and sacrificed at the appearance of neurologic symptoms. Brains were photographed under the fluorescence microscope, fixed over-night in 4% paraformaldehyde, cryoprotected in 20% sucrose, frozen and sectioned with a cryostat.

Cells transplantation into adult mouse brains were performed as previously described in [25] with the help of a stereotaxic table using the following coordinates from the Bregma: AP, 1.0 mm; L, 1.5 mm left and 2.5 mm below the skull surface. Animals were injected with 15000-100000 GFP-positive cells and then monitored daily for the appearance of neurological symptoms.

### Immunostaining

Immumostainings on brain sections or cultured cells were performed using the following antibodies: mouse monoclonal antibodies against Nestin (1:250; BD Pharmingen, San Diego, CA) and glial fibrillary acidic protein (GFAP, 1:200; Sigma); rabbit polyclonal antibodies against Olig2 (1:200; Sigma) and NG2 (1:300; Chemicon-Millipore, Billerica, MA); chicken polyclonal antisera against GFP (1:500; Abcam, Cambridge, United Kingdom); rat monoclonal antibody against PDGFR-alpha (1:100; BD Pharmingen) and CD31 (1:500; BD Pharmingen). Binding of primary antibodies was revealed with appropriate secondary Alexa Fluor 488 (1:500; Invitrogen), Dy-Light 488 or Dy-Light 549 (1:500; Jackson Immunoresearch, West Grove, PA) or Cy3 (1:100; Jackson Immunoresearch)-conjugated antibodies. Nuclei were stained with 4',6-diamidino-2-phenylindole (DAPI) solution (1 μg/ml; Sigma). Fluorescence images were analyzed with the ImageJ software (W.S. Rasband, ImageJ, US National Institutes of Health, Bethesda, MD; http://rsb.info.nih.gov/ij/, 1997-2007).

### Vessel density quantification

Blood vessels were identified by immunopositivity to the CD31 antigen. CD31-stained sections from three wild type and two NG2-KO PDGF-B induced gliomas were analyzed. For each glioma, two different sections were taken, including at least two microphotographs for each section. In each microphotograph, at least two regions of 0.06 mm^2 ^within the tumor were randomly selected to measure the area occupied by CD31 positive cells. This was done by using a threshold mask in the ImageJ software to circumscribe the immunopositive regions whose areas were measured by using the *wand *tool. Hence, for each section the percentage of CD31 positive area in the tumor were calculated as the ratio between the sum of CD31-positive areas and the sum of the areas of the analyzed regions. At this point, the average of the percentage of CD31 positive areas was calculated for each tumor and for the two tumor groups (wild type and NG2-KO).

### Quantitative PCR

RNA was extracted from two wild type and two NG2-KO PDGF-B induced glioma cultures with QIAzol reagent (Qiagen s.p.a., Milan, Italy) according to the manufacturer's guidelines. cDNA was then obtained from 500 ng of RNA using the iScript retrotranscription kit (Bio-Rad Laboratories, Hercules, CA). Quantitative real-time polymerase chain reaction (PCR) was performed on 1:100 of the retrotranscription reaction using iQ SYBRGreen Supermix (Bio-Rad Laboratories). PCR was performed in triplicate and repeated twice. PDGF-B mRNA quantifications were normalized to the housekeeping gene Rpl41 (NM_018860). The sequences of the primers are available on request.

### Western blot

Two NG2-KO and two wild type PDGF-B induced glioma cultures were harvested in lysis buffer containing: 50 mM HEPES (pH 7.5), 5 mM EDTA, 150 mM NaCl, 1% Triton^® ^X-100 detergent and protease inhibitors (Complete, Roche Applied Science). The level of PDGFR-alpha and phosphorylated PDGFR-alpha were previously normalized to alpha-tubulin, then the ratio between the activated and the total receptor was considered. Western blot was performed using the following antibodies: mouse anti-phospho PDGFR-alpha (1:1000; Gentaur Molecular Products, Bruxelles, Belgium), mouse anti-alpha-tubulin (1:5000, Sigma), rat anti-PDGFR-alpha (1:5000, BD Pharmingen), and the secondary anti-mouse (1:5000, Pierce) and anti-rat (1:5000, GE Healthcare) antibodies conjugated to HRP.

### Data analysis

Means and standard errors were calculated from different experiments. "n"; denotes the number of cells counted in the *in vitro *experiments or the number of animals used for *in vivo *experiments. "nExp" denotes the number of independent experiments performed. The threshold for statistical significance, which was determined with a 2-tailed Student's t-test, was considered as P < 0.05.

## Results

**Figure 1:**
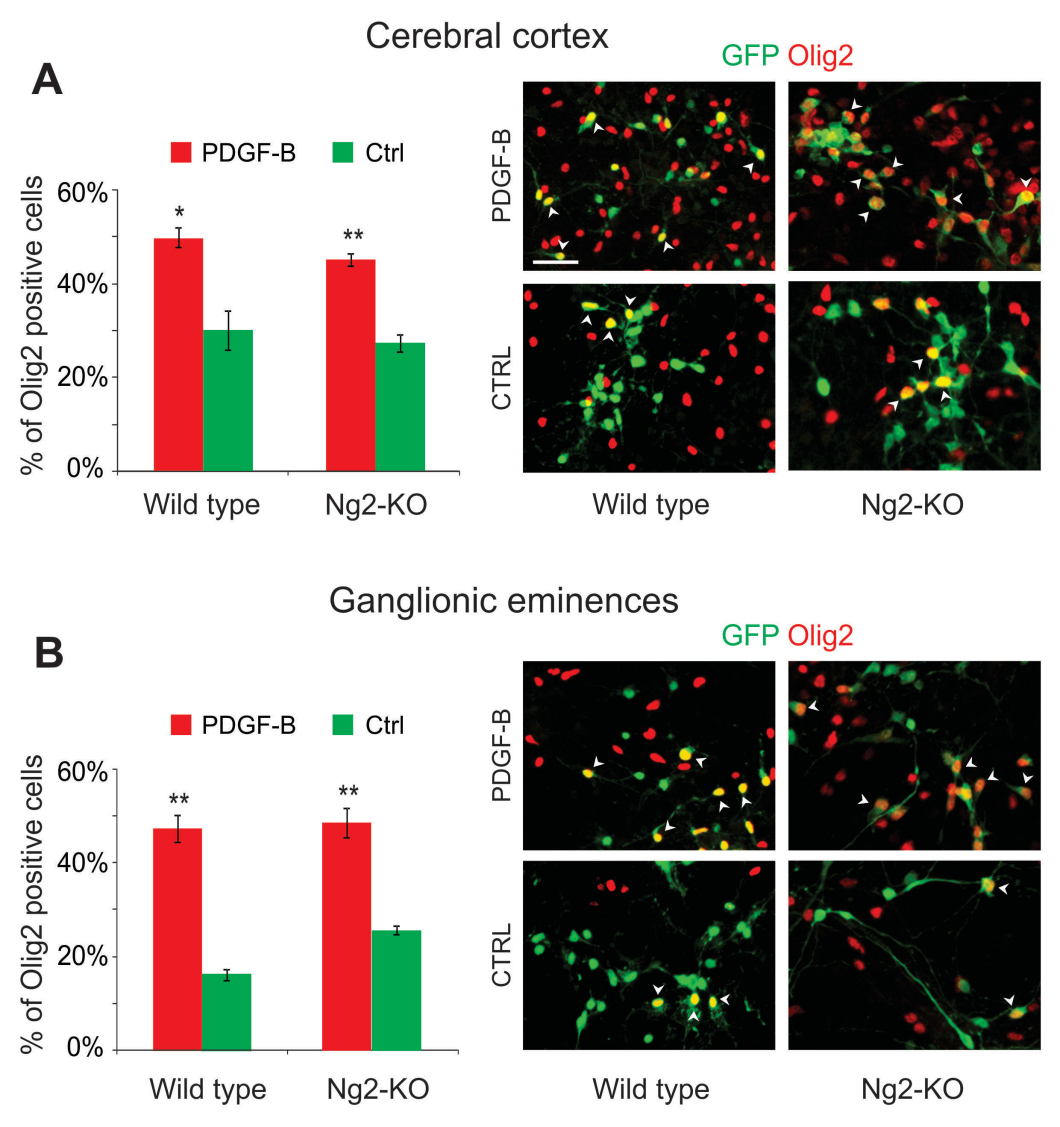
**PDGF-B transduction induces an oligodendroglial fate in cultured neural progenitor cells**. (A-B, left side) Histograms showing the percentage of Olig2-positive cells in PDGF-B (red bars) and control (green bars) transduced cortical (A) and GE (B) cultures obtained from wild type and NG2-KO embryos. (A-B, right side) Immunofluorescence micrographs of cortical (A) and GE (B) cultures derived from wild type and NG2-KO mice following the transduction with PDGF-B/GFP or control retroviruses. The immunoreactivity to Olig2 and GFP is shown in red and in green, respectively. NG2-KO cultures: nExp = 3; Wild type cultures: nExp = 2. *p < 0.05 and **p < 0.01 in a 2-tailed Student's t-test. Scale bar: 50 μm

We previously demonstrated that an early event following the retroviral overexpression of PDGF-B consists of the re-specification of cultured embryonic neural precursor cells to an oligodendroglial identity [23]. Based on the roles of NG2 in modulating PDGF signaling in different contexts [13-15,32], we decided to test if NG2 is required for PDGF-B to influence the fate of neural progenitor cells. We cultured E14 progenitor cells from the cerebral cortex of both NG2-KO and wild type mice and we infected them with high titre replication-deficient retroviruses coding for PDGF-B and the GFP fluorescent reporter (PDGF-B/GFP). Six days after infection, the cultures were immunostained for Olig2 to quantify the percentage of differentiating OPCs. The analysis showed that both wild-type and NG2-KO cultures responded in the same way to PDGF-B transduction. PDGF-B was able to significantly increase the percentage of Olig2-positive cells compared to cultures transduced with a control vector carrying only the reporter gene (Figure 1A). In particular, in the wild type, Olig2-positive cells raised from 30 ± 4% (n = 3500) to 50 ± 2% (n = 5300, nExp = 2, ttest p ≤ 0.05) and, in the NG2-KO cultures, from 27 ± 2% (n = 3900) to 45 ± 1% (n = 5100, nExp = 3, ttest p ≤ 0.01). Similar results were obtained with cultures derived from the ganglionic eminences (GE; Figure 1B).

The above data demonstrate that NG2 is not necessary for PDGF-B overexpression to induce an oligodendroglial fate in cultured neural progenitor cells.

**Figure 2:**
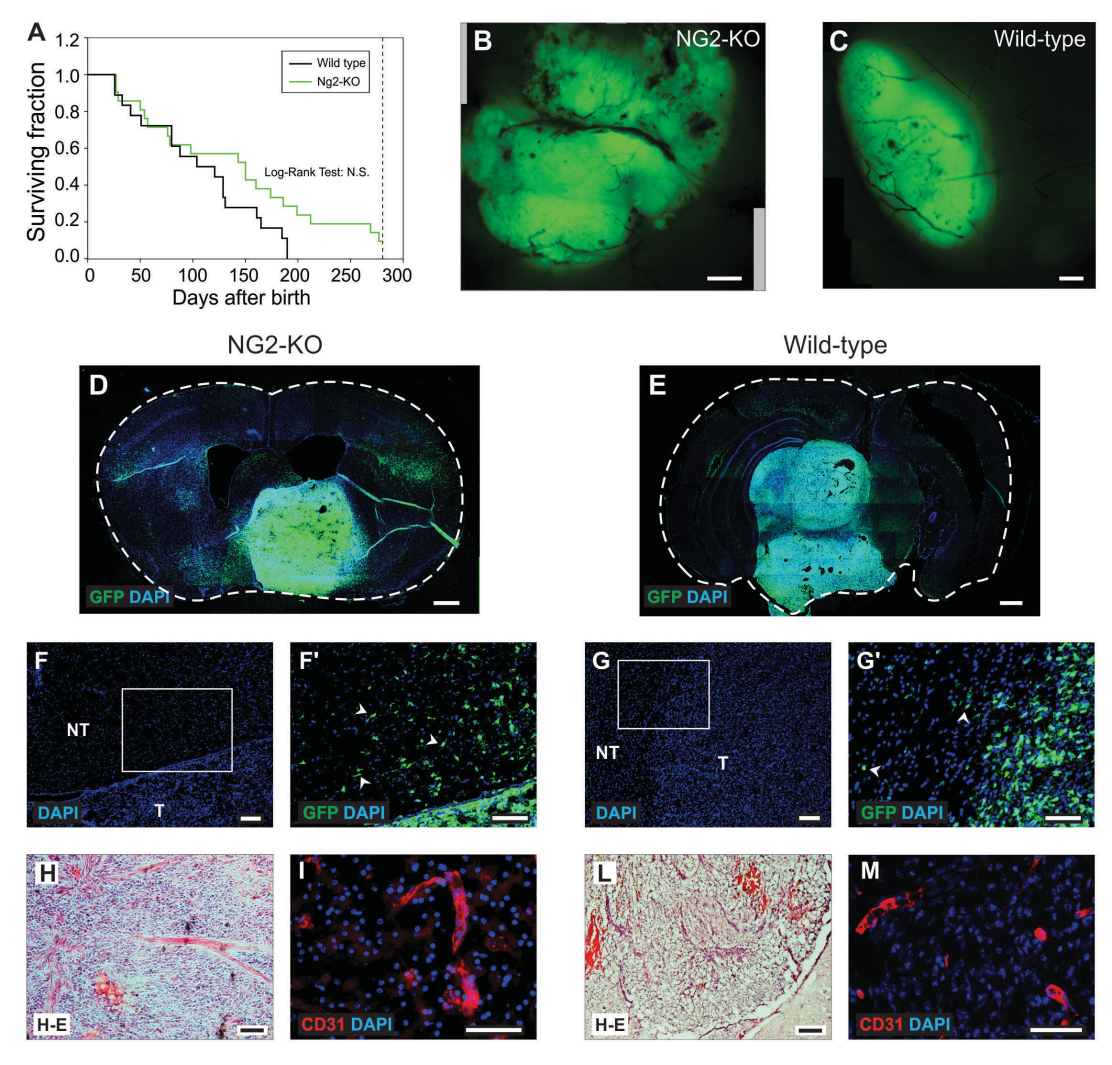
**PDGF-B overexpression induced gliomas in NG2-KO mice**. (A) Survival curves following PDGF-B embryonic transduction of NG2-KO (green line) and wild type (black line) mice. The Log-rank test performed on the survival curves of NG2-KO and wild type animals showed that there are no significant differences (Log-rank Test = N.S.) between the two distributions. (B-C) Fluorescence images of PDGF-B-induced tumors in NG2-KO (B) and wild type (C) mice. (D-M) Coronal sections of tumors obtained in NG2-KO (D, F, F', H, I) or wild type (E, G, G', L, M) mice stained with DAPI for nuclear staining in blue and antibodies for the indicated antigens (D-G', I, M) or with hematoxylin and eosin (H, L). F' and G' are magnifications of the insets in figures F and G respectively, arrowheads point infiltrating cells. NT: non-tumor zone; T: highly cellularized tumor zone. Scale bars: 0.5 mm (B-E); 100 μm (F-M).

Recently it has been proposed that NG2 may have a role in the induction of gliomas by PDGF-B [33], despite the fact that NG2 has been previously shown to specifically influence only PDGF-A signaling [13-16]. We therefore decided to clarify this issue by testing whether NG2 may affect PDGF-B-induced gliomagenesis in a model that we recently established, which is based on *in vivo *transduction of embryonic mouse neural progenitors [23,25]. Telencephalic progenitors of NG2-KO mouse embryos were transduced *in utero *at 14 days post coitum (dpc) with the PDGF-B/GFP expressing retroviral vector described above. After birth, NG2-KO injected animals were monitored daily for neurological symptoms (e.g. lethargy, ataxia, imbalanced stance and gait) which appeared in 90% of the animals (19 out of 21) within 280 days (Figure 2A). When explanted and observed under fluorescence microscope, the brains of all mice displaying symptoms showed wide masses of GFP-positive cells as observed in PDGF-B transduced wild type animals (Figure 2B-C). The survival of NG2-KO mice (n = 21) was compared to that of a group of wild type mice (n = 18) by applying the Log-rank test which showed that there is no difference (p = 0.09) in the survival of the two groups (Figure 2A). These data indicate that PDGF-B-driven gliomagenesis occurs normally in the absence of NG2.

Moreover, similarly to PDGF-B-induced tumors in wild type mice, NG2-KO gliomas showed all the typical features of high grade human gliomas, such as densely cellularized areas, highly vascularized regions and cells with a remarkable ability to infiltrate the healthy areas of the brain (Figure 2D-M). In particular, a quantitative analysis showed that there were no differences in the portion of tumor area occupied by blood vessels in NG2-KO (3.2 ± 0.1%) and wild type (3.3 ± 0.7%; p = 0.9) mice (Additional file 1). Furthermore, neither NG2-KO nor wild type showed any significant difference in the density of infiltrating cells: (454 ± 24 cells/mm^2^, n = 727 and 382 ± 86 cells/mm^2^, n= 567, respectively; p = 0.57).

However, it is important to note that PDGF-B induced gliomagenesis could occur in NG2-KO mice due to a compensatory event based on a higher activation of PDGFR-alpha or to a higher PDGF-B expression level. To rule out these possibilities we decided to quantify the level of phosphorylated PDGFR-alpha protein and of PDGF-B mRNA. As shown in Additional file 2A, despite some variability between tumors, a similar fraction of PDGFR-alpha protein was activated in wild type and NG2-KO tumor cells. Moreover, the expression level of transduced PDGF-B did not show any significant difference between the two animal groups (p = 0.8; Additional file 2B). From all these data we conclude that NG2 is not necessary for the formation of gliomas induced by PDGF-B overexpression, in agreement with previous data obtained in smooth muscle cells showing that PDGF-B signaling is not altered in NG2-KO mice [13].

**Figure 3:**
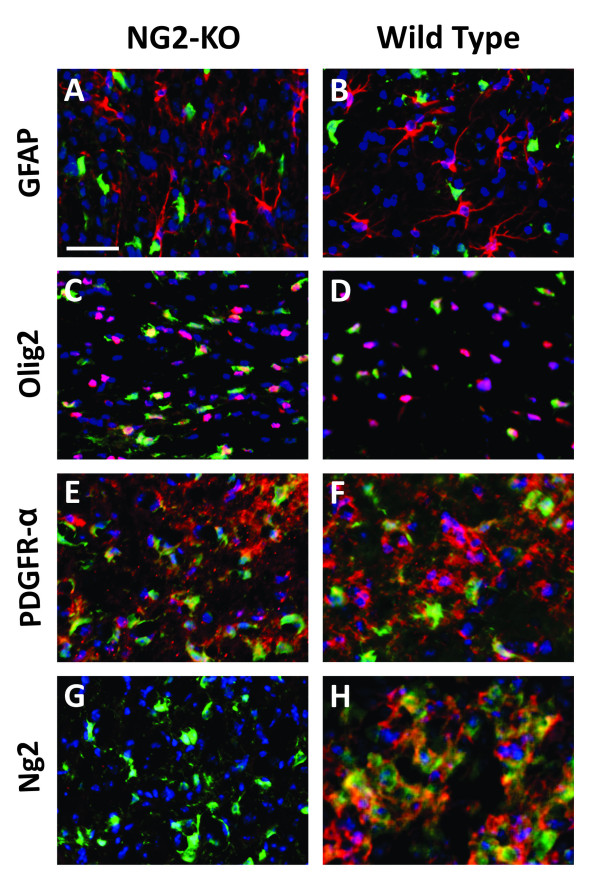
**PDGF-B overexpression induced oligodendrogliomas in NG2-KO mice**. (A-H) Immunofluorescence stainings of NG2-KO (A, C, E, G) and wild type (B, D, F, H) glioma sections with anti-GFP antibody in green, DAPI for nuclear staining in blue and antibodies for the indicated antigens in red. Scale bar: 50 μm

However, the above observations do not clarify whether NG2 has a role in the determination of the glioma subtype. We therefore analyzed tumors induced by PDGF-B in NG2-KO mice by assessing the expression of a panel of markers which allow distinguishing between different neural cell types. Analogously to tumors induced in wild-type mice, those induced in NG2-KOs did not express the astrocytic marker GFAP, even if GFAP-positive reactive astrocytes were frequently abundant within tumor masses (Figure 3A,B). On the contrary, PDGF-B-induced gliomas expressed high levels of oligodendrocyte progenitor markers such as the transcription factor Olig2 and the receptor PDGFR-alpha (Figure 3C-F). The only difference we found between tumor cells in NG2-KO and wild-type mice was, obviously, the expression of NG2 (Figure 3G,H), confirming the efficiency of the knockout. We therefore conclude that the lack of NG2 expression does not affect the ability of PDGF-B overexpression to induce tumors with oligodendroglial characteristics.

**Figure 4:**
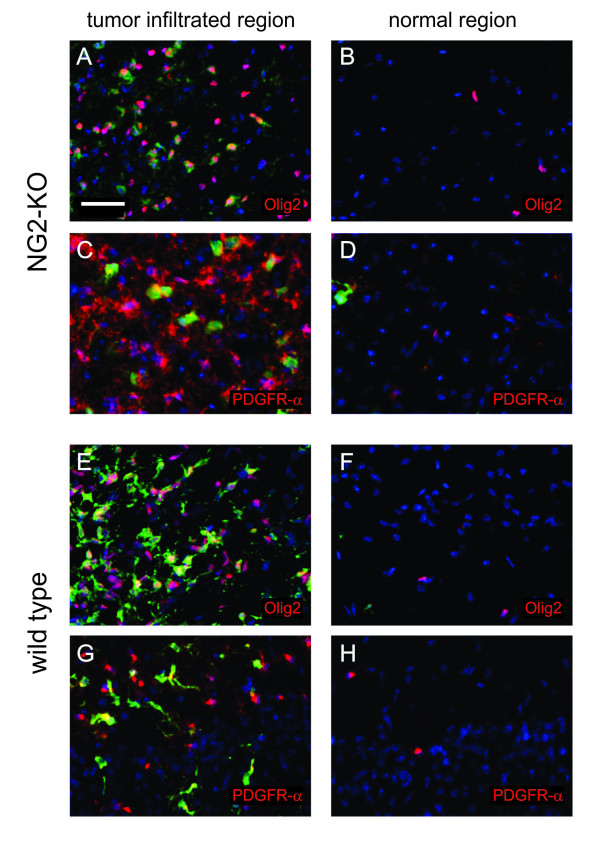
**PDGF-B-induced tumors recruit OPCs also in the absence of NG2 expression**. (A-H) Immunofluorescence stainings of NG2-KO (A-D) and wild type (E-H) glioma sections with anti-GFP antibody in green, DAPI for nuclear staining in blue and antibodies for the indicated antigens in red. The left column of the figure corresponds to tumor infiltrated regions, while pictures on the right represent normal brain regions next to tumor areas showing the basal level of OPC markers expression for comparison. Scale bars: 50 μm

In primary PDGF-B-induced gliomas generated in wild type animals, there are abundant untransduced (GFP-negative) OPCs [23,24,36]. These cells are recruited to the tumor mass by paracrine effects of PDGF and/or other glioma-secreted factors and likely contribute to the tumors high growth rate. We therefore evaluated presence and identity of GFP-negative cells within primary NG2-KO tumors. Similarly to wild type PDGF-B-induced tumors, NG2-KO gliomas harbored an abundant population of recruited OPCs, as revealed by the expression of Olig2 and PDGFR-alpha by the GFP-negative cells in the tumor infiltrated brain regions (Figure 4). These findings suggest that resident OPC recruitment occurs normally in NG2-KO gliomas, showing that NG2 is not necessary for the paracrine recruitment of resident OPCs by PDGF-B-induced gliomas.

**Figure 5:**
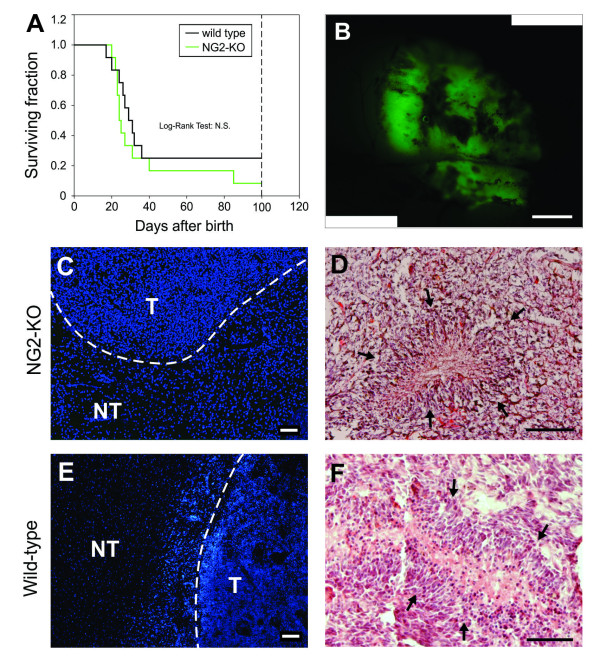
**Cells derived from PDGF-B-induced gliomas are tumorigenic also in the absence of NG2 expression**. (A) Survival curves following transplantation into adult brains of PDGF-B-induced tumor cells derived from wild type (black line) or NG2-KO (green line) mice. The Log-rank test performed on the two survival distributions showed that there are no significant differences (Log-rank Test = N.S.). (B) Fluorescence image of a NG2-KO secondary tumor. (C-F) Coronal sections of secondary tumors derived from NG2-KO (C-D) or wild type (E-F) mice stained with DAPI (C, E) or hematoxylin and eosin (D, F). NT: non-tumor zone; T: highly cellularized tumor zone; Dashed contours highlight the tumor mass; arrows point pseudopalisade structures. Scale bars: 1 mm (B); 100 μm (C-F).

We then evaluated if gliomas induced by PDGF-B overexpression in NG2-KO mice maintain the ability to propagate as tumors after *in vivo *transplantation, as is typical of those from wild type mice [25]. To this end, we transplanted PDGF-B expressing glioma cells from 3 independent NG2-KO tumors into adult mouse brains. Recipient mice were of either wild-type or NG2-KO genotype. As shown in Figure 5A-B, 11 out of 12 recipients developed secondary tumors within 85 days after transplantation. This frequency was compared, with the Log-rank test, to the tumor occurrence observed when wild type PDGF-B-induced glioma cells were injected in wild type recipients (9 mice out of 12; p > 0.05), revealing that NG2-KO glioma cells are able to give rise to secondary tumors with high frequency, indistinguishably from wild type tumors. In the above mentioned experiment survival data for wild-type and NG2-KO recipients were pooled, since no significant difference was observed between the two groups (Additional file 3).

The histological analysis of the secondary tumors derived from NG2-KO primary gliomas revealed large, highly cellularized, compact masses with pseudopalisades surrounding necrotic areas (Figure 5C-D). These observations demonstrate that secondary tumors from NG2-KO mice are indistinguishable from those obtained in wild type mice (Figure 5E-F) and possess a high tumorigenic potential.

**Figure 6:**
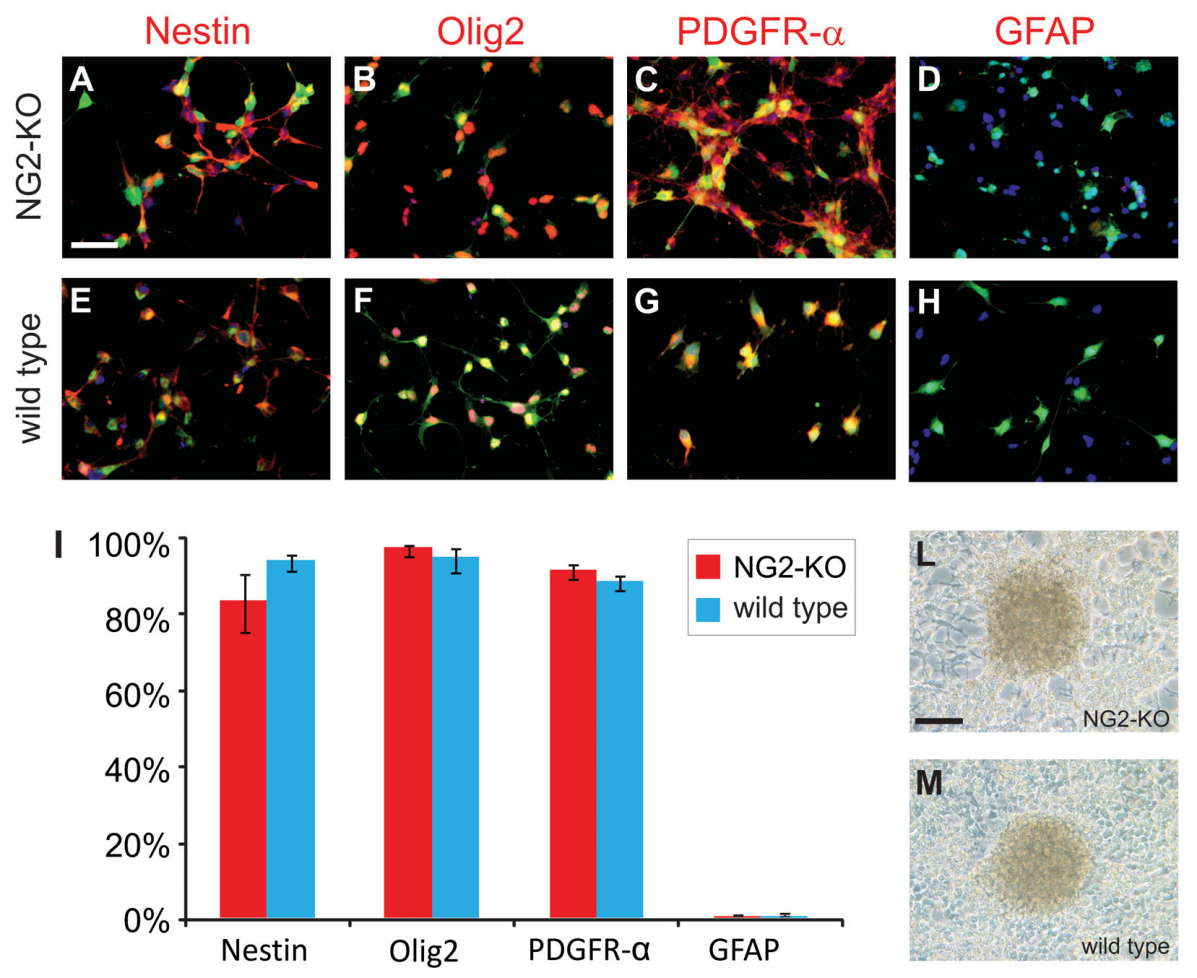
**PDGF-B-induced tumors can be propagated in culture also in the absence of NG2 expression **.(A-H) Immunofluorescence stainings of NG2-KO (A-D) and wild type (E-H) glioma cultures with anti-GFP antibody in green, DAPI for nuclear staining in blue and the antibody for the indicated antigen in red. (I) Histogram showing the percentage of cells expressing the indicated markers in NG2-KO (red bars) and wild type (blue bars) glioma cell cultures. (L-M) Bright field microphotographs of NG2-KO (L) and wild type (M) glioma cells showing the ability to form foci in vitro. Scale bars: 50 μm (A-D); 100 μm (L-M).

Another feature of PDGF-B-induced tumors is their capability to be cultured *in vitro *maintaining the phenotypic and tumorigenic characteristics of the original tumor [23,25]. We therefore tested this ability on 3 independent NG2-KO PDGF-B-induced tumors, and observed that 2 of them could be maintained *in vitro*. The immunocytochemical analysis showed that these cells, similarly to their wild type counterparts expressed Nestin, a marker for neural progenitor cells, the OPC markers Olig2 and PDGFR-alpha, whereas they were immuno-negative to the astrocytic marker GFAP (Figure 6A-I).

We have recently reported that a hallmark of highly malignant PDGF-B-induced gliomas is their *in vitro *ability to override cell-cell contact-mediated inhibition of proliferation [25]. We therefore evaluated the sensitivity of NG2-KO glioma cells to increased cell density in culture, and we observed that, like wild type cells, NG2-KO tumor cells bypassed cell-cell contact inhibition and maintained their proliferative activity, eventually forming three dimensional cell foci (Figure 6L-M). Moreover, a quantitative analysis performed by counting the density of foci showed that there are no significant differences in the ability to form foci between NG2-KO and wild type glioma cells (see Additional file 4 as example of the data obtained from this experiment). Altogether these data clearly show that NG2 is not necessary to maintain the phenotypic characteristics of cultured PDGF-B-induced gliomas.

**Figure 7:**
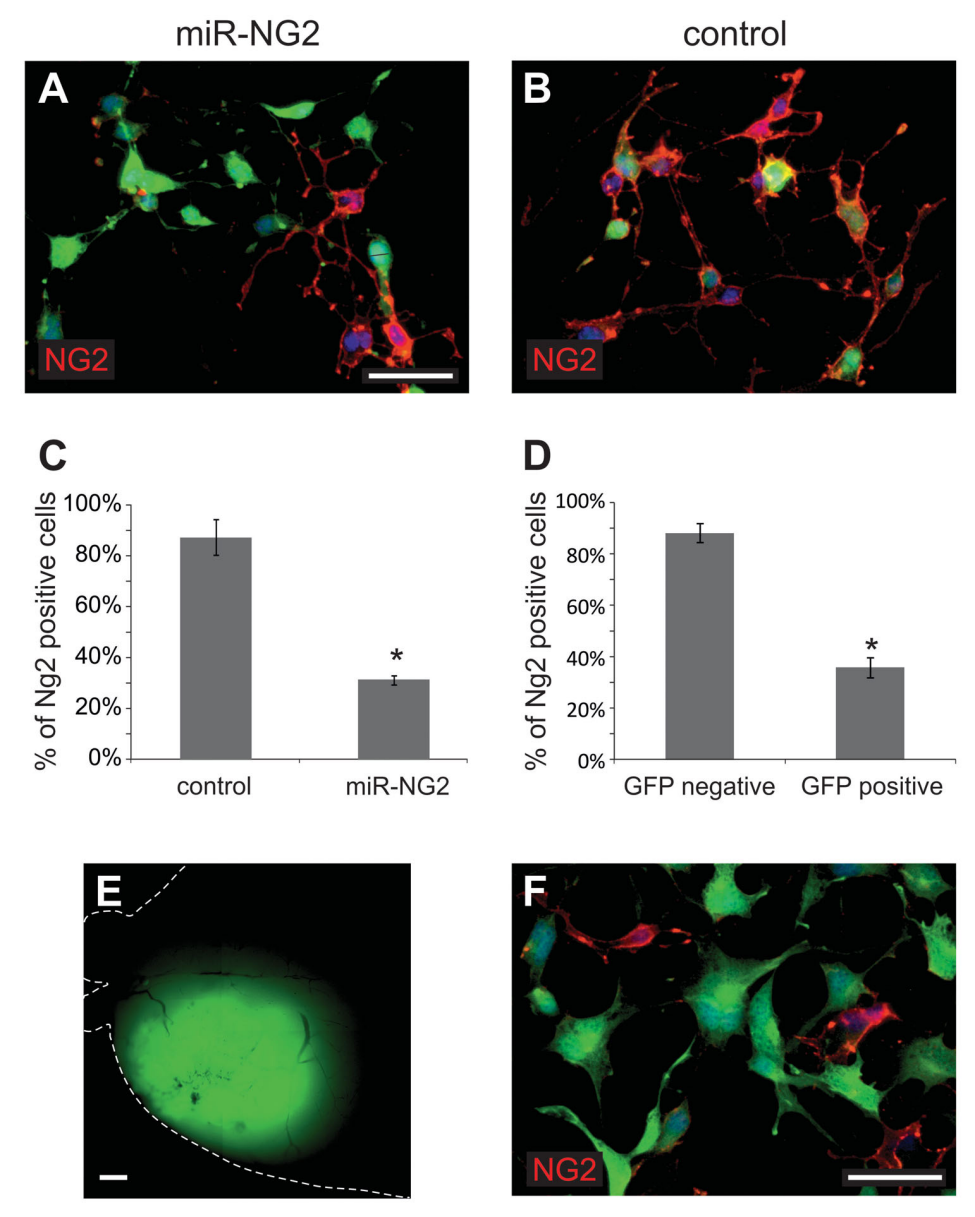
**NG2 silencing does not impair the tumorigenic potential of cells derived from PDGF-B-induced tumors**. (A-B) Immunofluorescence stainings of PDGF-B-induced glioma cultures transduced with miR-NG2 (A) or miRneg (B) and stained with anti-GFP antibody in green, anti-NG2 antibody in red and DAPI (nuclei) in blue. (C-D) Histograms showing the efficiency of miR-NG2 silencing in PDGF-B-induced glioma cultures (C) or in cells extracted from a secondary tumor generated by the intracranial injection of PDGF-B-induced glioma cells previously transduced with miR-NG2 (miR-NG2 secondary tumor; D). GFP positive cells represent glioma cells which were actually transduced with miR-NG2, while GFP-negative cells were not transduced. (E) Fluorescence image of a miR-NG2 secondary tumor. (F) Immunofluorescence staining of miR-NG2 secondary tumor cells with anti-GFP antibody in green, anti-NG2 antibody in red and DAPI (nuclei) in blue. Scale bars: 50 μm (A-B, F); 0.5 mm (E).

However, all the above observations do not exclude the possibility that compensatory effects, which may occur in NG2-KO embryos, could mask the actual role of NG2 during the development of a glioma in a normal context. We therefore analyzed this issue by using an engineered microRNA (miRNA-NG2) to silence NG2 expression in wild type PDGF-B induced glioma cells. To test the silencing efficiency of the miRNA-NG2, we transduced glioma cells, already expressing a PDGF-B/DS Red vector, with retroviral vectors expressing GFP and either the miRNA-NG2 or a negative control miRNA (miRneg, not complementary to any cellular mRNA) and, after 7 days, we compared the expression of NG2 in the two conditions. As shown in Figure 7A-C, the transduction of the miRNA-NG2 significantly reduced NG2 expression from 88 ± 7% (n = 671) of control cells, to 32 ± 2% (n = 865, nExp = 2, ttest p < 0.05).

To evaluate the possibility that NG2 may be necessary for the tumor-propagating potential of PDGF-B induced gliomas, we transduced cells from two independent gliomas with the miRNA-NG2, the miRneg or a vector carrying only GFP and we injected them into the brains of adult mice (miRNA-NG2: n = 6; miRneg: n = 9 ; control GFP: n = 18). Since the retroviral infections never resulted in the transduction of all cells, we injected mixed populations of transduced and untransduced cells. We reasoned that if NG2 silencing exerted negative effects on the tumorigenic potential of these cells, this would result in the formation of GFP-depleted secondary tumors. The use of mixed populations allowed us to have an internal control of gliomagenesis that eliminated the doubt about any technical problem which may occur during injection. Moreover, to avoid stochastic cell population effects from depleting secondary tumors of the GFP-positive cell fractions, we only injected glioma populations containing at least 40% GFP-positive cells. After injection, no significant difference in the percentage of GFP-positive secondary tumors was shown between mice injected with miRNA-NG2- (67%) and control- (67%) transduced cells (Table 1).

**Table 1:** NG2 silencing did not prevent tumorigenesis driven by PDGF-B induced glioma cells.

	miR-NG2	control
GFP-positive tumors	66.7%	66.7%

GFP-negative tumors	16.7%	14.8%

not analyzed *	0%	7.4%

no tumor **	16.7%	11.1%

The percentage of tumors for the indicated classes were shown. * Tumors were not analyzed because the mice were found already dead and the fluorescent signal was no more reliable due to autofluorescence. ** Mice that did not develop a tumor.

Since not all the cells expressing an engineered miRNA usually reach an adequate level of gene silencing, we quantified the percentage of NG2 expressing cells in one miRNA-NG2 infected secondary glioma to rule out the possibility that GFP-positive secondary tumors would exclusively derive from cells maintaining NG2 expression. As shown in Figure 7D-F, the level of NG2 silencing in the secondary glioma cells is analogous to that previously shown in vitro (Figure 7C). The percentage of cells expressing NG2 decreased from 88 ± 2% of GFP-negative cells (n = 176) to 36 ± 2% of GFP-positive cells (n = 235).

All those data demonstrate that PDGF-B induced gliomagenesis can occur after the abrogation of NG2 expression, thus raising serious doubts on the efficacy of NG2 targeting as therapy against gliomas.

## Discussion

The observation that human gliomas often highly express NG2 suggested that this molecule could play an important role in gliomagenesis [2,5,33,37]. This view is corroborated by some preliminary data [33] and by the observation that NG2 expression is important for some OPCs to correctly proliferate during development [32] and to respond to PDGF signaling [13,15]. Here, however, we showed that NG2 expression is not necessary for the induction of OPC lineage features mediated by PDGF-B overexpression, thus questioning the role of NG2 for oligodendrocyte lineage determination.

Moreover, we tested the relevance of NG2 during PDGF-B-induced gliomagenesis. Our results clearly show that NG2 is dispensable for the formation of gliomas following the overexpression of PDGF-B and that tumors induced in NG2-KO animals are indistinguishable from those observed in wild type mice. This suggests that tumor development occurred analogously in the two contexts. The similarities between NG2-KO and wild type gliomas include the presence of highly infiltrating cells, a typical feature of human gliomas, and vascularized areas which ultimately require the induction of angiogenesis, indicating that in this context this process is not significantly influenced by the lack of NG2. Importantly, the absence of NG2 did not significantly affect the ability of gliomas to be maintained for long term in culture, to override cell-cell contact inhibition of proliferation forming cell foci in vitro and to regenerate a glioma after orthotopic transplantation, all features representing functional proxies for high malignancy [25].

Moreover, the use of an engineered miRNA directed against NG2 in wild type glioma cells strengthens our results, eliminating the possibility that glioma formation in NG2-KO mice could be due to compensatory events taking place in these animals. Our data also confirm previous observations performed in different experimental systems [13] showing that NG2 is not able to bind PDGF-B to influence PDGF-B-driven signaling, while they reduce the expectations on the NG2 proteoglycan as a target molecule useful to efficiently treat gliomas. While our data show that NG2 is dispensable for PDGF-B induced gliomagenesis, other authors reported preliminary data from a somehow different system (PDGF-B transduction in the adult white matter), pointing instead to a role for NG2 in this same process [33]. Lower transformation efficiencies (only 4 out of 11 wild type mice developed a glioma), and as yet small experimental numbers, may have contributed to this discrepancy, and a full report will be necessary for further hypotheses to be drawn.

It is furthermore to be noted that since NG2 seems to influence specifically PDGF-A-driven signaling and PDGF-A can also induce the formation of gliomas *in vivo *[22], NG2 could have a therapeutic role in PDGF-A overexpressing gliomas. Moreover, since NG2 is highly expressed in human gliomas, it may be possible to use it as a tag allowing the delivery of therapeutic molecules to glioma cells.

## Conclusions

We conclude that, unexpectedly, the lack of NG2 does not prevent the generation of malignant gliomas driven by PDGF-B overexpression. Moreover, an accurate comparison of gliomas induced by PDGF-B in NG2-KO and wild type mice did not reveal any significant difference in terms of tumor development, histopathology, molecular marker expression, ability to propagate in vitro and to regenerate tumors after orthotopic in vivo transplantation.

Questioning the potential role of NG2 in glioma formation, our data reduce the expectations on the proteoglycan NG2 as a target molecule for the treatment of this kind of tumor. We therefore think that our findings are important to redirect the scientific research involved in the development of new glioma therapies.

## Abbreviations

DAPI: 4',6-diamidino-2-phenylindole; GE: ganglionic eminences; GFAP: glial fibrillar acid protein; GFP: green fluorescent protein; OPCs: oligodendrocyte progenitor cells; KO: knockout

## Competing interests

The authors declare that they have no competing interests.

## Authors' contributions

MT and IA planned and carried out most of the experiments and drafted the manuscript. FC planned and carried out part of the experiments and critically revised the manuscript draft. RP helped to provide the NG2-KO strains. ET carried out the embryonic injections and helped to acquire the data. PM conceived and planned the experiments and critically revised the manuscript draft. All authors read and approved the final manuscript.

## Pre-publication history

The pre-publication history for this paper can be accessed here:

http://www.biomedcentral.com/1471-2407/10/550/prepub

## Supplementary Material

Additional file 1 **Lack of NG2 expression does not impair vessels formation in PDGF-B-induced tumors**. Histogram shows the percentage of tumor area occupied by vessels (identified by immuno-positivity to CD31 marker) in wild type and NG2-KO PDGF-B-induced gliomas. Click here for file

Additional file 2 **The level of PDGFR-alpha activation and of PDGF-B expression is similar in wild type and NG2-KO glioma cells**. (A) Western blot of two wild-type (WT) and two NG2-KO (KO) PDGF-B-induced glioma cultures for the indicated proteins. The ratio between the level of activated PDGFR-alpha and total PDGFR-alpha is shown in the histogram. Values were normalized to the WT-1 culture. (B) Histogram shows the levels of PDGF-B mRNA relative to the housekeeping gene Rpl41 in wild type and NG2-KO glioma cultures. Values represent the means of real-time PCR quantifications for two independent cultures per condition. Click here for file

Additional file 3 **Cells derived from PDGF-B-induced gliomas generated in NG2-KO mice are tumorigenic**. Survival curves following injection of PDGF-B induced tumor cells derived from NG2-KO mice into NG2-KO (blue line) or wild type (red line) recipients. Click here for file

Additional file 4 **Cells derived from PDGF-B-induced tumors form foci in vitro also in the absence of NG2 expression**. Histogram shows the average number of foci in the analyzed area (4 mm^2^) for the wild type and the NG2-KO glioma cultures (p = 0.7). Click here for file
