# Supplementary figures and images for: PDGF-B-driven gliomagenesis can occur in the absence of the proteoglycan NG2
Source: BMC Cancer. 2010 Oct 12;10:550. doi: 10.1186/1471-2407-10-550 (PMC2964636; doi:10.1186/1471-2407-10-550)

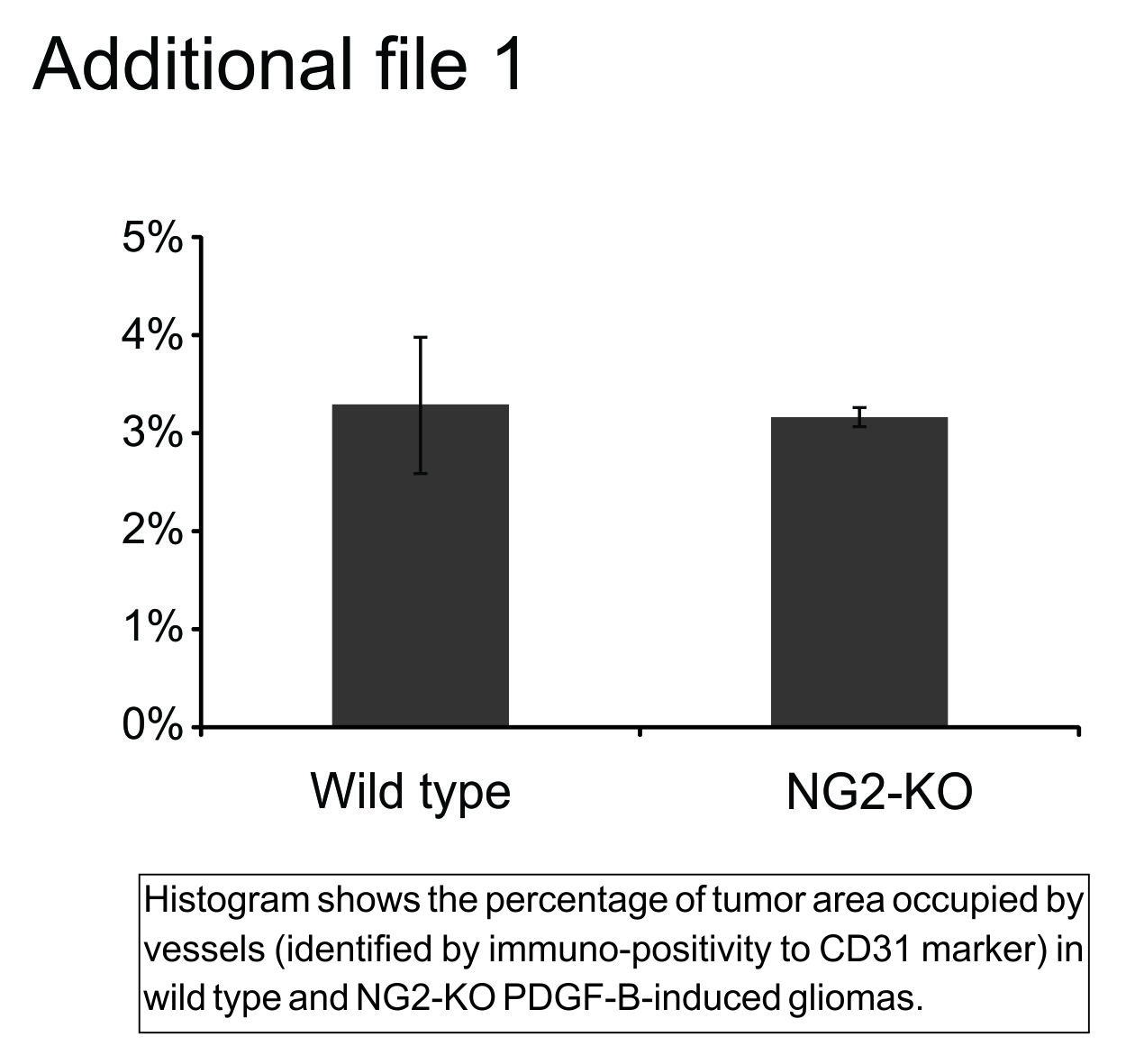

Supplement: Additional file 1 — Lack of NG2 expression does not impair vessels formation in PDGF-B-induced tumors. Histogram shows the percentage of tumor area occupied by vessels (identified by immuno-positivity to CD31 marker) in wild type and NG2-KO PDGF-B-induced gliomas. [file 1471-2407-10-550-S1.JPEG]

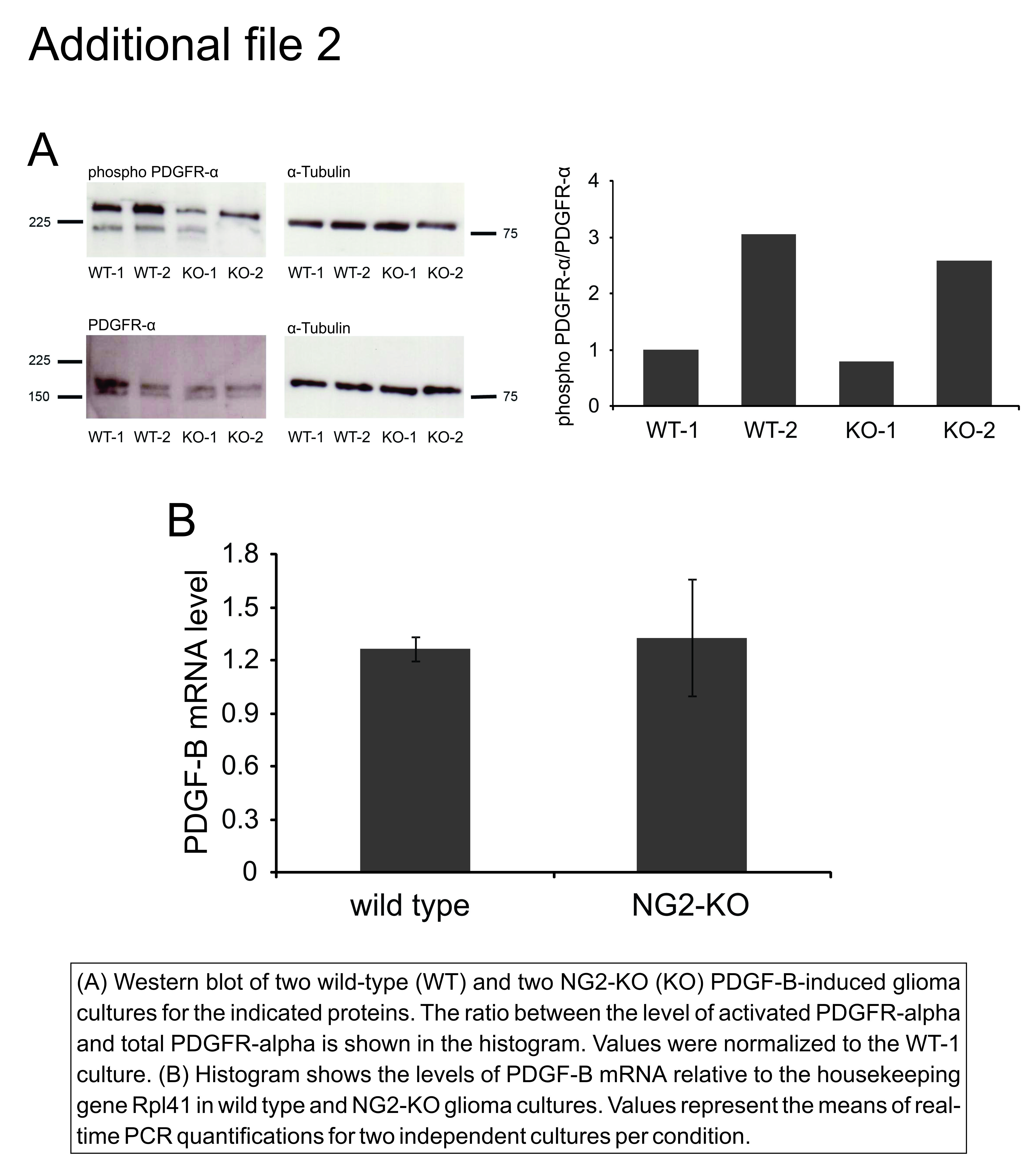

Supplement: Additional file 2 — The level of PDGFR-alpha activation and of PDGF-B expression is similar in wild type and NG2-KO glioma cells. (A) Western blot of two wild-type (WT) and two NG2-KO (KO) PDGF-B-induced glioma cultures for the indicated proteins. The ratio between the level of activated PDGFR-alpha and total PDGFR-alpha is shown in the histogram. Values were normalized to the WT-1 culture. (B) Histogram shows the levels of PDGF-B mRNA relative to the housekeeping gene Rpl41 in wild type and NG2-KO glioma cultures. Values represent the means of real-time PCR quantifications for two independent cultures per condition. [file 1471-2407-10-550-S2.JPEG]

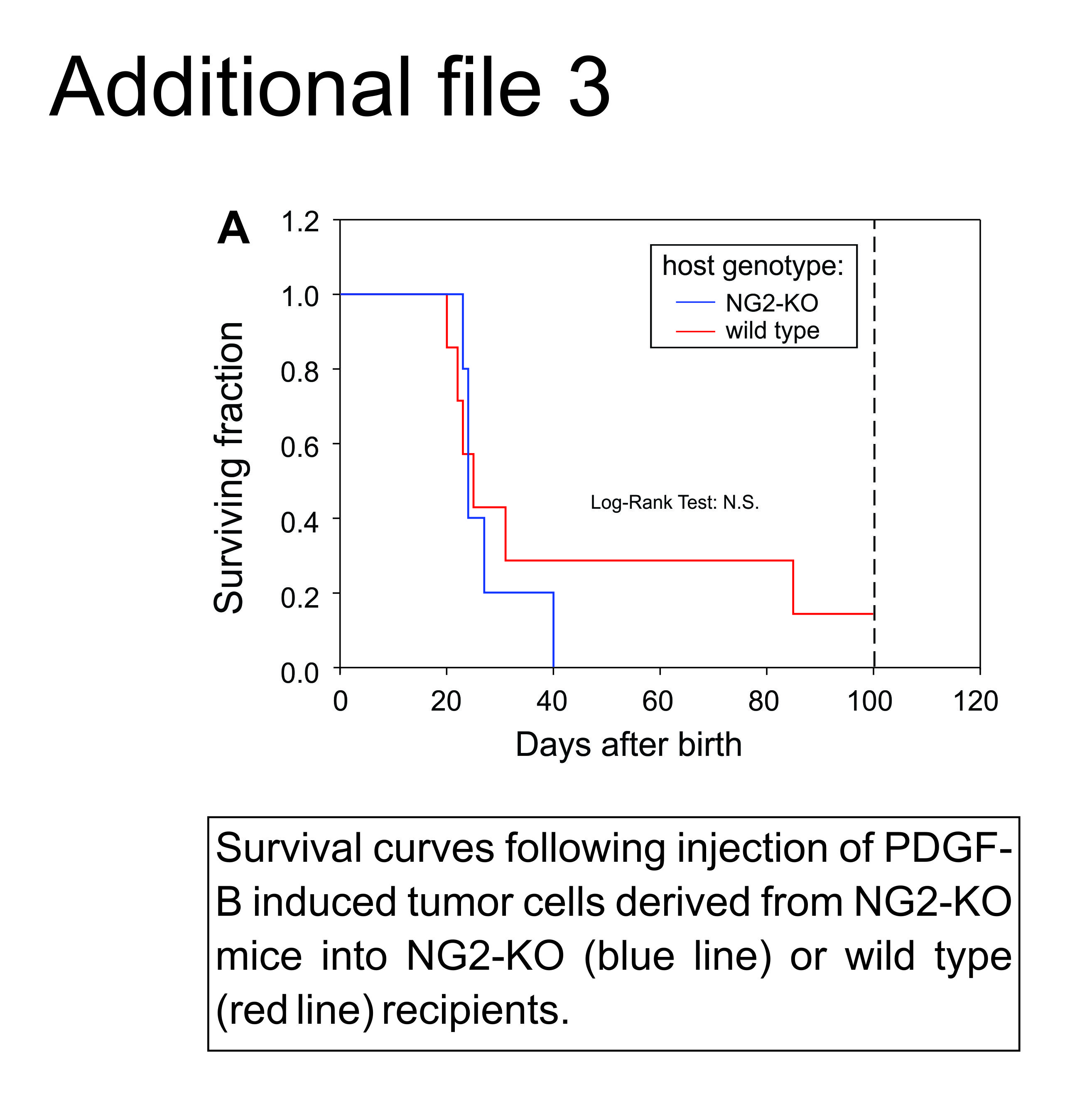

Supplement: Additional file 3 — Cells derived from PDGF-B-induced gliomas generated in NG2-KO mice are tumorigenic. Survival curves following injection of PDGF-B induced tumor cells derived from NG2-KO mice into NG2-KO (blue line) or wild type (red line) recipients. [file 1471-2407-10-550-S3.JPEG]

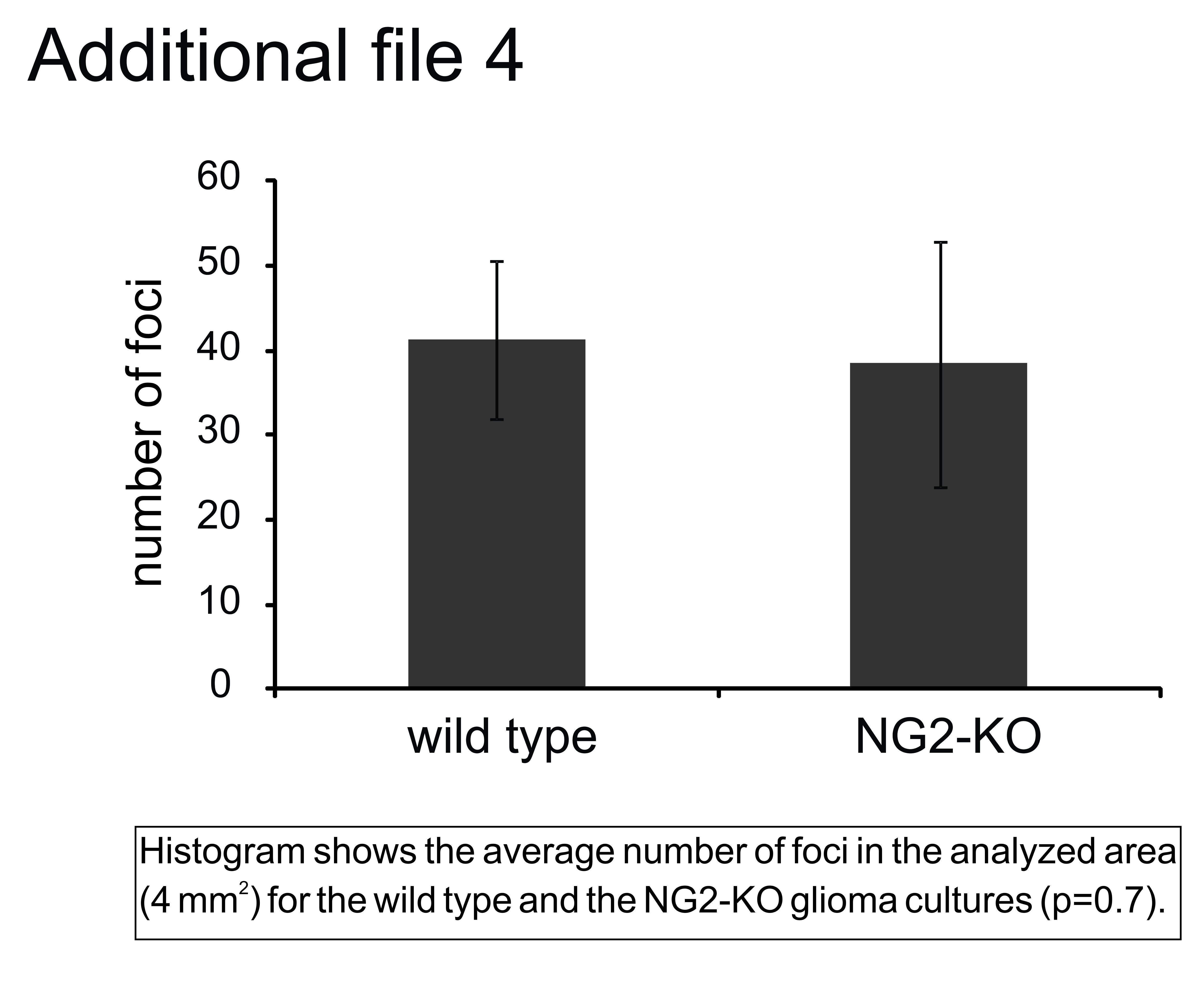

Supplement: Additional file 4 — Cells derived from PDGF-B-induced tumors form foci in vitro also in the absence of NG2 expression. Histogram shows the average number of foci in the analyzed area (4 mm2) for the wild type and the NG2-KO glioma cultures (p = 0.7). [file 1471-2407-10-550-S4.JPEG]
